# Supplementary material for: Are carbon emissions trading and green financial instruments synergistic? -Comprehensive quantitative research based on content analysis
Source: PLoS One. 2024 Mar 7;19(3):e0298601. doi: 10.1371/journal.pone.0298601 (PMC10919601; doi:10.1371/journal.pone.0298601)
Supplement: S1 Data — (ZIP) [file pone.0298601.s001.zip › Data/地级市Z_F工作报告.zip!/╣π╬≈/░┘╔1⁄2╩╨/└┤╫╘╣1⁄2╓┌║┼í╛┬φ┐╦╩2╛▌═°í┐_2014.docx]

一、2013年工作回顾

　　——预计全年地区生产总值同比增长9%，扭转了经济持续回落的态势；

　　——财政收入首次突破100亿元；

　　——城镇居民人均纯收入、农民人均纯收入增幅高于全区平均水平；

　　——左右江革命老区振兴规划启动并被提到国家层面；

　　——成功开通百色至北京果蔬绿色专列；

　　——成功开通百色至越南高平国际道路客货运输线路；

　　——成功开通百色靖西至越南高平边境旅游线路；

　　——成功实施“田七回家”工程；

　　——全面启动百东新区建设；

　　——启动实施“旅游业赶超跨越三年行动计划”；

　　——创建国家卫生城市通过国家级技术评估；

　　——首次通过国家科技进步考核；

　　——百色灌区项目规划报告完成水利部专家技术咨询；

　　——扶贫开发连续两年年度考核排全区第一；

　　——植树造林面积连续3年排全区第一；

　　——社会稳定动态七项工作和公众安全感指标排名全区前列。

　　一年来，统筹推进稳增长、调结构、促改革、惠民生，各项工作取得了明显成效：

　　（一）经济实力稳步提升。预计全市实现地区生产总值828亿元，同比增长9%。其中第一产业完成146.7亿元，同比增长7%；第二产业完成456.3亿元，同比增长10%；第三产业完成225亿元，同比增长9%。全社会固定资产投资820亿元。财政收入完成107.7亿元，同比增长9.8%。年末金融机构存款余额758亿元、贷款余额592亿元，分别增长12.1%和14.7%。

　　（二）转型升级稳中提质。三次产业结构由上年的18.5：55.5：26调整为17.8：55：27.2。铝基和锰基新材料、非冶金铝、节能环保型金属、高端装备制造、碳酸钙、林木加工、电子信息等新产业加快发展。规模以上万元工业增加值能耗同比下降5.3%，万元工业增加值水耗同比下降7.03%。新增非公企业3275户、个体工商户1236户。

　　（三）扶贫攻坚成效显著。新一轮扶贫攻坚战、兴边富民行动和“整村推进”扶贫开发扎实有效。设立了“曾宪梓产业扶贫专项担保资金”，投入财政产业扶贫资金1.05亿元。完成广州市帮扶的12个“整村推进”示范村建设任务，与广州市的扶贫协作向产业合作转变。完成扶贫培训5.3万人。投入财政专项扶贫资金2.8亿元，实施一批水、电、路项目，贫困村基础设施明显改善。预计全市减贫20万人，贫困发生率下降5个百分点。

　　（四）交通基础设施不断完善。预计交通领域完成投资140亿元，增长36%。百色至靖西、靖西至那坡、马山至平果、崇左至靖西4条高速公路以及云桂铁路加快建设。河池至百色、乐业至百色高速公路、黄桶至百色铁路、南昆铁路二线以及百色巴马新机场前期工作加快推进。国道、省道路网16个改造项目和“百村通畅工程” 192个项目深入实施。鱼梁航运枢纽船闸实现通航。

　　（五）工业经济平稳增长。预计全部工业总产值1116亿元，同比增长12%；规模以上工业增加值350亿元，同比增长11%。工业投资完成285亿元。生态型铝产业示范基地建设加快，全市铝产业完成总产值600亿元，同比增长24%。工业园区完成工业总产值875亿元，同比增长14%。全市新增亿元以上企业9家，新增规模以上企业41家。

（六）特色农业取得突破。预计完成农林牧渔业总产值237亿元，增长7.3%。粮食产量117.3万吨，超额完成全年任务。秋冬农业开发总面积超过300万亩，其中秋冬菜面积162万亩。糖料蔗、蔬菜、水果、茶叶、蚕茧、肉类等产量、产值实现双增长。渔业产值增长23.5%，增幅排全区第一位。林下养鸡肉鸡出栏1.3亿羽，增长25.9%。实施“田七回家”工程，落实种植面积2.07万亩。新增市级农业重点龙头企业9家、农民专业合作社264家。无公害农产品、绿色食品、有机农产品基地认证突破200万亩，其中有机农产品基地4.74万亩，排全区第一位。乐业县成为全国5个有机农业示范县之一。田东获批国家现代农业示范区农业改革与建设试点县。

　　（七）现代服务业较快发展。全市接待旅游总人数1678.6万人次，增长23.3%；实现旅游总收入123.8亿元，增长28.6%。全年公路客货运周转量分别增长7.1%和15.1%。邮政普遍服务营业收入、快递业务收入均突破1亿元。新建商品房销售面积增长 21.6%。新增小额贷款公司6家。完成社会消费品零售总额178亿元，增长13.5%。

　　（八）城镇建设稳步推进。加快提升老城区、完善新城区、挺进开发百东新区，做美扮靓右江。百色大道、迎龙大道、体育沙滩公园等项目开工建设。园博园、龙景区路网等项目加快推进。市体育中心一期、科技文化中心等项目竣工。中心城区面积突破40平方公里，主骨架路网基本形成。各县城镇基础设施进一步完善。城乡清洁工程、城乡风貌改造、名镇名村建设、村镇规划集中行动继续推进。田阳、田林、西林县荣获 “广西园林城市”称号，乐业、隆林、西林荣获“广西卫生县城”称号。全市城建领域投资完成210亿元。城镇化率达39.5%，比上年增加2个百分点。

　　（九）改革创新扎实推进。全国农村改革与建设试点工作扎实推进，农村金融改革取得新成果，产权交易市场建设、土地流转经营、农村产权确认等制度进一步完善。国有企业改革稳步推进。组建百矿集团。制约生态型铝产业发展的瓶颈问题在企业改革和体制创新上开始破题。田阳金叶纸业公司成为中国造纸业第一家在上海股权托管交易中心挂牌的企业。百色电力公司成为广西首家完成私募债备案发行工作的企业。市县共建产业园区稳步推进。集体林权制度配套改革取得新成效。农民家庭承包耕地流转步伐加快。扩权强县工作深入开展。

　　（十）开放合作再上新台阶。积极融入南北钦防玉崇百区域经济一体化，与河池市、崇左市签订跨市战略合作框架协议。与广州市、云南文山州、贵州黔西南州在产业园区建设、能源、旅游等方面开展跨省合作。参与“美丽广西港澳行”活动，推动民企对接，与上海交大、上海知名企业交流合作取得良好成效。与越南等东盟国家开放合作不断深化，百色沿边开发开放试验区、中国龙邦—越南茶岭跨境经济合作区建设扎实推进。招商引资到位资金560.6亿元，增长4%。对外贸易进出口总额5.75亿美元，增长13%。

　　（十一）生态品牌更加凸显。万元GDP能耗同比下降3.7%，主要污染物排放量削减目标基本完成。完成植树造林57.47万亩，森林覆盖率达66.62%。环境安全隐患大清查大整治全面开展。资源综合利用和矿区生态复垦取得新成效。平果铝采矿用地改革作为全国唯一试点，通过国家验收。田东县成为广西唯一的“国家循环经济示范县”。凌云县荣获“中国长寿之乡”称号。城市污水集中处理率达83.28%、生活垃圾无害化处理率达100%、环境空气质量优良率达95%以上。新增耕地面积618.5公顷。“美丽百色·清洁乡村”活动全面开展，乡村环境极大改善。

　　（十二）民生保障坚实有力。全市涉及民生支出168.92亿元，占财政支出比重70.21%，同比增长11.56%。城镇居民可支配收入21517元，增长10%，农民人均纯收入5418元，增长13.5%，分别高于全区1个和1.5个百分点。城镇新增就业2.24万人，农村劳动力转移就业新增5.7万人。居民消费价格指数增长2.5%，控制在5%的增长目标内。65.23 万人享受城乡低保补助，实现应保尽保。新型农村和城镇居民社会养老保险参保率97.64%。五项社会保险参保人数增长7.4%。新农合参合率达98.68%。参加农房政策性保险86.86万户。开工建设保障性住房11626套、竣工9164套。完成农村危房改造2.41万户。实施农村饮水安全工程1421处。十项为民办实事工程顺利完成。创新型百色建设全面启动。7个县区通过国家科技进步考核，田阳县荣获“全国科技进步先进县”。实施教育项目单项工程1921个，29.4万名学生享受农村义务教育阶段学生营养补助。成立百色市教育基金会。高考总上线率连续4年排在全区前两位。全市180个基层医疗卫生机构和1358所村卫生室全部实施国家基本药物制度，194个中央投资卫生项目竣工。全国人口计生综合改革示范市建设取得新进展，人口自然增长率6.98％。。提前完成“十二五”村村通广播电视工程项目建设。建成206个村级公共服务中心，公共文化基础设施场所免费开放工作全面推开。成功举办百色市第三届运动会。
